# Vindoline—A Natural Product from Catharanthus Roseus Reduces Hyperlipidemia and Renal Pathophysiology in Experimental Type 2 Diabetes

**DOI:** 10.3390/biomedicines7030059

**Published:** 2019-08-13

**Authors:** Oluwafemi Omoniyi Oguntibeju, Yapo Aboua, Mediline Goboza

**Affiliations:** 1 Department of Biomedical Sciences, Phytomedicine and Phytochemistry Research Group, Oxidative Stress Research Centre, Faculty of Health & Wellness Sciences, Cape Peninsula University of Technology P.O. Box 1906, Bellville 7535, South Africa; 2 Department of Health Sciences, Faculty of Health and Applied Sciences, Namibia University of Science and Technology, Windhoek 13388, Namibia

**Keywords:** cardiovascular diseases, diabetic kidney disease, oxidative stress, inflammation, apoptosis

## Abstract

Cardiovascular diseases (CVDs) and kidney diseases in diabetes are linked to increased mortality and morbidity. The aim of this study was to evaluate the effect of vindoline derived from *Catharanthus roseus* in diabetes-induced CVDs and kidney disease through assessing inflammation, oxidative stress, hyperlipidaemia and kidney function parameters. Type 2 diabetes was induced in male Wistar rats by 10% fructose water intake for two weeks, followed by a single intraperitoneal injection of 40 mg/kg body weight of streptozotocin (STZ). Six groups (*n* = 8) of randomly divided rats received vindoline (20 mg/kg) or glibenclamide (5 mg/kg) daily for 6 weeks via oral gavage. Lipid profile markers and markers of atherogenic index were decreased in diabetic rats after treatment with vindoline and glibenclamide. The levels of urea were significantly increased in the diabetic control group (13.66 ± 0.9) compared to the diabetic groups treated with vindoline and glibenclamide (10.62 ± 0.6 and 10.82 ± 0.8), respectively. Vindoline did not significantly alter the levels of inflammatory cytokines; however glibenclamide lowered the levels of TNF-α in kidney and heart tissues. Vindoline improved the ferric reducing antioxidant power in diabetic hearts, while superoxide dismutase (SOD) oxygen radical absorbance capacity was increased in the kidneys. Lipid peroxidation was reduced when compared to the diabetic controls. Vindoline restored the structure of the renal parenchyma and was accompanied by significant decrease in the expression of caspase 9 in diabetic rats when compared to the diabetic controls.

## 1. Introduction

The global prevalence of cardiovascular diseases (CVDs) is a major health concern that is rising substantially [1]. Recent statistical projections reported that 17 million deaths (31% of all global deaths) that occurred in 2015 were related to CVDs. It is disturbing to note that the prevalence of CVDs is mostly predominant in low- and middle-income countries where more than 75% of these CVDs-related deaths are currently documented [2]. It is well ascertained that unhealthy behavioural and lifestyle factors greatly influence the pathogenesis of CVDs [3]. Several studies have shown the negative roles that unhealthy dietary practices play in the development of a constellation of metabolic diseases such as diabetes mellitus. Therefore, a relationship between CVDs and type 2 diabetes mellitus has been established owing to the common risk factors shared [4]. It has also been shown that patients with uncontrolled hyperglycaemia are at 2–3 times greater risk of developing CVDs [5]. This is because diabetic patients exhibit dyslipidaemia; a metabolic condition characterised by hypercholesterolaemia, hyperlipidaemia and hypertriglyceridemia [3]. As time progresses, these abnormal lipids may accumulate in the walls of blood vessels causing the formation of plaque deposits that will then impede blood flow [6].

Increased blood glucose levels in DM have been shown to influence the pathogenesis of microvascular and macrovascular complications. Pathological effects of DM affect normal functions of kidneys, heart, blood vessels, nerves and the eyes and may eventually lead to death [3]. A hyperglycaemic environment encourages the persistent build-up of reactive oxygen/ nitrogen species (ROS/RNS) at the expense of antioxidant pools [7]. When this happens, an imbalance eventually occurs, leading to the development of oxidative stress (OS), which propagates the formation of diabetic complications [5]. In addition to OS, inflammation and insulin resistance play major roles in cellular events that drive the development of the complications [8]. Enhanced concentration of ROS accelerates oxidation and glycation of low-density lipoproteins (LDL). As a result, the modified LDL activates proinflammatory processes [9], resulting in the reduction of nitric oxide (NO) and increased vascular lipid deposition thus initiating endothelial dysfunction and overt atherosclerotic injury [10].

Cardiovascular disease and diabetic nephropathy (DN) are comorbidities that occur in type 2 diabetes mellitus (T2DM) [11]. Scientific studies have documented the role lipids play in the induction of glomerular and tubulointerstitial injury [12]. It is believed that the interrelationship observed among the mechanisms that drive dyslipidaemia, OS and inflammation are well involved in the pathogenesis of DN [13]. For that reason, lipid lowering treatments could be important in the prevention and treatment of diabetic nephropathy.

In order to control T2DM, different conventional treatment regimens have been put in place. These comprise drugs that suppress postprandial hyperglycaemia, possess insulotropic effects, lower hepatic glucose output and increase tissue sensitivity to insulin [14,15]. Concomitantly, as these drugs effectively lower blood glucose level, undesired effects are observed in diabetic patients [16]. Moreover, the unavailability of these orthodox drugs especially in poor rural settings calls for a continuous quest to identify possible alternative therapies that are safer and effective in the mitigation of T2DM and its complications. Medicinal plants may therefore serve as leads in the discovery of novel, safer and cheaper therapies for T2DM [4]. Their effects have been attributed to the presence of multiple biomolecules that work singly or synergistically to ameliorate hyperglycaemia, hyperlipidaemia, oxidative stress, inflammation and insulin resistance [17].

*Catharanthus roseus* is a plant that has been utilised since ancient times to treat and manage various ailments such as DM [18]. *C. roseus* is a rich source of indole alkaloids. Vincristine and vinblastine are indole alkaloids isolated from *C. roseus* that have been successfully incorporated into anticancer therapy [18]. Vindoline is one of the alkaloids that is highly concentrated in the leaves or twigs of the *C. roseus* [19]. In a previous study, administration of vindoline in rats resulted in hypoglycaemic effects [20]. It was then hypothesised that vindoline could be one of the major compounds that is responsible for the antidiabetic activities of *C. roseus* [20]. Yao and colleagues [19] reported that vindoline’s mode of antidiabetic action could be linked to β cell stimulation. However, the effect of vindoline on T2DM-induced oxidative stress and inflammation in the kidney and heart tissues has not yet been reported. Therefore, we investigated the effects of vindoline on possible markers of CVDs, oxidative stress and inflammation in a T2DM rat model that exhibits both insulin resistance and insufficiency.

## 2. Materials and Methods

### 2.1. Chemicals

Chemicals and reagents used were of analytical grade and purchased from Sigma-Aldrich (Johannesburg, South Africa). These include streptozotocin (STZ), 6-hydroxydopamine, 2-thiobabituric acid (TBA), malondialdehyde, 2,2′-Azobis(2-amidinopropane) dihydrochloride (AAPH), -6-Hydroxy-2,5,7,8-tetramethylchromane-2-carboxylic acid (Trolox), 2,4,6-tripyridyl-s-triazine (TPTZ) and ascorbic acid.

### 2.2. Bioactive Compound

The alkaloid vindoline was purchased from the company Best of Chemicals (BOC) Sciences, USA. Specific guidelines, storage and preparation methods were followed as per the supplier’s instructions.

### 2.3. Animal Handling and Ethics Statement

Animal ethical and experimental approval was sorted and granted by two committees: Ethics Committee for Research on Animals of the South African Medical Research Council (REF-01/17 approved on 29 March 2017) and from the Faculty of Health and Wellness Research Ethics Committee of Cape Peninsula University of Technology, South Africa (CPUT/HW-REC 2016/A4 approved on 23 August 2016).

The experimental animals used in this study were six weeks old (190–230 g) male Wistar rats procured from Charles River (Margate, United Kingdom). Two rats were housed per cage in temperature and humidity controlled standard environmental conditions with 12 h light–darkness cycle. All the rats were allowed to have free access to water and standard laboratory diet throughout the entire study period.

### 2.4. Animal Grouping

A total of forty-eight (48) animals were randomly divided into 6 groups of 8 rats: NC: nontreated normal control; NV: nondiabetic control treated with vindoline (20 mg/kg) body weight (b.w); NG: nondiabetic control treated with the standard antidiabetic drug glibenclamide (5 mg/kg b.w); DC: diabetic nontreated control; DV: diabetic treated with vindoline (20 mg/kg b.w); DG: diabetic treated with glibenclamide (5 mg/kg b.w).

### 2.5. Induction of Type 2 Diabetes Mellitus (T2DM)

To induce insulin resistance, diabetic groups were allowed to free access to 10% fructose water solution for 2 weeks instead of drinking normal tap water. In contrast, nondiabetic groups had free access to normal tap water. After this period, a single low dose of STZ (40 mg/kg b.w) prepared freshly in 0.1M citrate buffer (pH 4.5) was injected intraperitoneally after an overnight fast into the DC, DV and DG groups. A low dose of STZ induces partial β cell destruction. The nondiabetic groups NC, NV and NG were injected only with the same volume of 0.1M citrate buffer. Diabetes was confirmed 3 days after STZ induction. Rats were fasted for 4 h and thereafter fasting blood glucose was measured. Rats that had blood glucose concentrations greater than 15 mmol/L were considered diabetic.

### 2.6. Treatment

Daily treatment (for 6 weeks) through oral gavage with respective compounds (vindoline/glibenclamide) commenced after 5 days of STZ administration to ensure stable hyperglycaemia.

### 2.7. Oral Glucose Tolerance Test (OGTT)

An OGTT was conducted on day 28 in fasted rats, where rats receive their daily treatments. After 30 min a single dose of D-glucose (50%) solution (0.5 g/kg b.w) was administered orally to each animal. Subsequent blood glucose levels were measured at 0 (just before treatment with vindoline/glibenclamide), 30, 60, 90 and 120 min after the ingestion of glucose. Blood (10 microlitres) was collected through the tail of the rat by using tail prick method and glucose levels were measured using a glucometer.

### 2.8. Collection of Heart, Kidney and Blood Samples

At the end of the experiment, rats were fasted for 4 h, anaesthetised and terminated by using isoflurane gas at 2% with 1% oxygen during laparotomy. Blood was immediately drawn through the abdominal vena cava and collected into respective tubes. Tubes were allowed to stand for an hour at room temperature before centrifugation. The kidneys and hearts were removed and washed in cold phosphate buffered saline (PBS). Thereafter, the organs were weighed and dried using a paper towel. The left kidneys and hearts meant antioxidant analysis were snap frozen in liquid nitrogen and stored at −80°C for future analysis. The right kidneys were fixed in 10% (*v*/*v*) neutral buffered formalin and embedded in paraffin wax for histological and immunohistological analysis. Later, the snap frozen samples were homogenised in ice-cold respective buffers for different endogenous antioxidant activity determination. Homogenates were centrifuged for 15 min at 15,000 rpm at 4 °C, aliquoted and stored at −80 °C until analysis.

### 2.9. Relative Kidney and Heart Weights

The relative weights of the organs were calculated using the following formula; Relative organ weight = (organ weight ÷ total body weight) 100 g.

### 2.10. Serum Lipid Profile Measurement

Serum lipids such as total cholesterol (TC), triglycerides (TG) and high-density lipoprotein-cholesterol (HDL-c) were analysed by an Automated Chemistry Analyser: ABX PENTRA 400. Low density lipoprotein-c (LDL-c) LDL-c was calculated from TC, HDL-c and TG levels using the Friedewald’s equation: *TC-HDL-C-TG/5*.

Very low-density lipoprotein-c (VLDL-c) using the formula *TG/5*.

#### Atherogenic Indices

The extent of atherosclerotic plaques in the blood vessels was determined by assessment of the atherogenic indices serum (AIS). The atherogenic index is calculated using the following formulas.

- AIS = TC-HDL/HDL [21]
- AIS = TC/HDL [22]

### 2.11. Endogenous Antioxidant Analysis

The antioxidant enzyme activities of superoxide dismutase (SOD) and catalase (CAT) were determined in clear 96-well plate using the Multiskan plate reader (Thermo Fisher Scientific, Waltham, Massachusetts, USA). Spectrophotometric determination of SOD and CAT activity were measured in tissue homogenates following the modified method of Ellerby & Bredesen [23].

### 2.12. Lipid Peroxidation

Lipid peroxidation (LPO) in the kidney tissue was assessed by measuring the amount of malondialdehyde (MDA). MDA levels were determined by the thiobarbituric acid reactive substance (TBARS) method. The coloured complex formed when MDA reacted with thiobarbituric acid (TBA) was measured at 532 nm according to the method of Tug et al. [24].

### 2.13. The Oxygen Radical Absorbance Capacity (ORAC)

The ability of biological samples to prevent oxidation of a fluorescein reagent by the peroxyl radical of 2,2’-Azobis(2-amidinopropane) dihydrochloride (AAPH). In the presence of antioxidants, hydrogen atoms are donated to the peroxyl radical, hence inhibiting degradation of the fluorescein reagent. The net area under the curve depicts the fluorescence intensity and therefore the amount of antioxidant present in the sample. Fluorescence intensity was measured by Fluoroskan at every minute over 120 min by excitation and emission at 485 nm and 538 nm respectively [25].

### 2.14. Ferric Reducing Antioxidant Power (FRAP)

The ferric reducing antioxidant power of tissue samples was measured using the method that was developed by Benzi and Strain in 1996 [26]. FRAP is a concentration independent colorimetric assay that assesses the strength of samples to reduce the ferric ion (Fe^3+^) to ferrous ion (Fe^2+^). Reduction of the Fe^3+^ ion present in the (TPTZ) complex to a ferrous form (tripyridyl triazine complex) results in blue coloured product. Absorbances were determined using a spectrophotometer at a wavelength of 593 nm.

### 2.15. Inflammatory Cytokines Measurement

The levels of both proinflammatory (interleukin-10) and anti-inflammatory cytokines (TNF-α and IL-1β,) were detected using a Bio-plex Pro Magnetic bead-based Luminex kit (Merck, Billerica, MA, USA) on the Bio-Plex platform. 200 mg of the tissue was homogenised in phosphate buffer (pH 7.4), centrifuged at 14,000× *g* for 15 min at a temperature of 4 °C. The undiluted supernatants were mixed with dyed magnetic beads coated with primary anticytokine antibodies. The primary antibodies then bind to its corresponding cytokine. This results in the formation of complex which in turn binds to biotinylated anticytokine secondary antibody in a sandwich manner. The reaction mixture is detected by the addition of fluorescent streptavidin–phycoerythrin (streptavidin–PE), which binds to the biotinylated detection antibodies allowing visualization and analysis by Bio-Plex Manager software.

### 2.16. Histological Assessment of the Kidney Using Haematoxylin and Eosin Stain

After sacrificing, the right kidneys were fixed in 10% (*v*/*v*) neutral buffered formalin and dehydrated by passing through increasing concentrations of alcohol, cleared and embedded in paraffin blocks. Blocks were cut to produce 5-µm-thick sections that were mounted on a slide. The paraffin in tissue sections were removed by passing them through xylene, decreasing concentrations of alcohol and finally in water. The sections were then stained in haematoxylin and eosin stain.

### 2.17. Immunohistochemistry Analysis

Apoptotic markers such as caspase 3, caspase 9 and Bcl-2 were assessed using immunohistochemistry (Leica Bond autostainer (Leica Biosystems, South Africa). Kidney sections that were fixed in formalin and embedded in paraffin were first deparaffinised before the antigen retrieval process. Specific antibodies of the selected apoptotic markers were added to the slides with exposed antigen sites. The peroxidase block was added to the slides in order to avoid nonspecific binding. Thereafter, primary antibodies were added to the slides, incubated using the postprimary antibody at room temperature for 30 min. 3,3′ Diaminobenzidine (DAB) a chromogen solution and DAB substrate buffer polymer were introduced to the slides to facilitate the production of a brown end product. Slides were then counterstained using haematoxylin in a 5 min incubation step. Kidney tissue sections were dehydrated by moving slides in a series of graded alcohols followed by a mounting step using dibutyl phthalate xylene (DPX). Slides were viewed and images captured using the EVOS XL Cell imaging microscope. Positive intensities were analysed and quantified using ImageJ Immuno Profiler software (version 10.2 image analysis).

### 2.18. Statistical Analysis

Results were analysed using GRAPH PAD Prism software package, Version 5.0. Data were expressed as mean ± standard error mean (SEM). The comparisons within groups were determined by using the one-way analysis of variance (ANOVA) and Bonferroni’s multiple test comparison. The values were considered to be statistically significant when the *p*-value was < 0.05.

## 3. Results

### 3.1. Effect of Vindoline on the 2 h OGTT in Nondiabetic and Diabetic Rats

**Figure 1:**
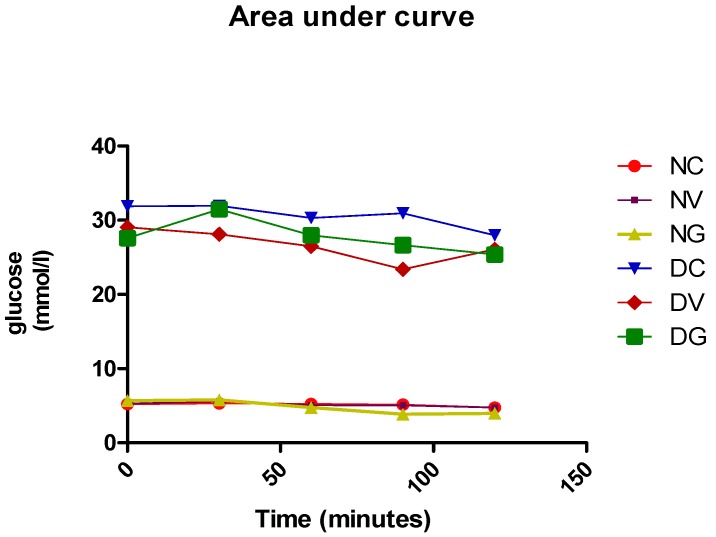
Graphical presentation of the effect of vindoline in nondiabetic and diabetic rats on 2-h oral glucose tolerance test. Data represented as means ± SEM. NC: normal control; NV: normal control treated with vindoline; NG: normal control treated with glibenclamide; DC: diabetic control; DV: diabetic treated with vindoline; DG: diabetic treated with glibenclamide.

The blood glucose levels of nondiabetic rats, diabetic control rats and diabetic rats treated with vindoline or glibenclamide is shown in Table 1 and Figure 1 below. Significant elevated blood glucose levels were observed in all T2DM-induced groups when compared to the normal control group. At 1.5 h post-glucose load the OGTT results clearly indicate the significant reduction in blood glucose level in the diabetic group treated with vindoline (from 29.05 ± 2.41 to 23.39 ± 2.4 mmol/L) when compared to the untreated diabetic controls (from 31.89 ± 0.75 to 30.96 ± 0.81 mmol/L). Pretreatment of diabetic rats with vindoline or glibenclamide reduced the area under curve (AUC) relative to the diabetic control group by 15% and 9%, respectively. No significant differences in glucose levels were observed in the nondiabetic treated groups when compared to the nondiabetic nontreated control group.

### 3.2. Effect of Vindoline Administration on the Kidney and Heart Weights and Kidney Function Parameters in Nondiabetic and Diabetic Rats

The changes in heart and kidney weights of the control and experimental groups are represented in Table 2 below. Significant organ hypertrophy in all T2DM-induced rats was observed when compared to the nondiabetic rats. Administration of vindoline or glibenclamide did not significantly prevent organ hypertrophy in diabetic rats when compared to the diabetic control group. On the other hand, normal rats treated with vindoline (NV) showed no significant changes in both organs when compared to the normal control group NC.

In comparison to the untreated diabetic control group, diabetic rats that were treated with vindoline and glibenclamide showed significantly reduced levels of serum urea although the recorded concentrations were significantly higher when compared to all nondiabetic groups.

Additionally, the serum creatinine concentration levels were significantly reduced in the diabetic groups that were treated with vindoline and glibenclamide (37.24 ± 1.6 mg/dl and 37.49 ± 2.3 mg/dl; *p* < 0.05), respectively, when compared to the untreated diabetic controls (47.59 ± 4.3 mg/dl). When compared to the normal control group (33.72 ± 1.5 mg/dl), only the diabetic control group recorded significantly elevated serum creatinine levels (*p* < 0.05).

### 3.3. Serum Lipid Levels in Normal and T2DM-Induced Rats after Receiving Respective Treatments for 6 Weeks

Table 3 below indicates the level of serum lipids in nondiabetic and T2DM-induced rats after respective treatment interventions. Significantly higher levels of lipids were observed in the untreated diabetic control group when compared to all the groups (*p* < 0.05). Both treatment of diabetic rats with vindoline or glibenclamide reinstated the serum lipid levels to near normal, as shown in Table 3 below, since there were no significant differences in the levels of ‘bad lipids’ when compared to the normal control group. Moreover, administration of vindoline to diabetic rats (DV) significantly increased the levels of the ‘good’ cholesterol: HDL which was 65.58 ± 1.02% of the total cholesterol, whereas the diabetic control group had the lowest percentage of the HDL (51.85 ± 3.86%, *p* < 0.05).

**Figure 2:**
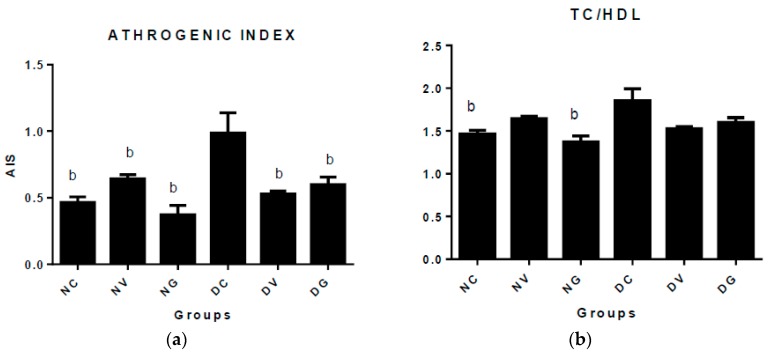
Atherogenic index assessment after 6-week treatment period (**a**,**b**). Data represented as means ± SEM. ^b^ *p* < 0.05 vs. diabetic control. NC: normal control; NV: normal control treated with vindoline; NG: normal control treated with glibenclamide; DC: diabetic control; DV: diabetic treated with vindoline; DG: diabetic treated with glibenclamide.

The atherogenic index was determined to evaluate the potential risk of CVDs development in the diabetic rats. As shown in Figure 2, the intake of vindoline and glibenclamide in diabetic rats reduced the risk of future CVDs development as both products significantly reduced the atherogenic index when compared to the untreated diabetic controls. The diabetic control group exhibited increased values of atherogenic index when compared to all treatment groups (*p* < 0.05). Likewise, the ratio of serum TC/HDL in the untreated diabetic control group increased significantly when compared to the normal control group (*p* < 0.05), while the diabetic groups that was treated with vindoline and glibenclamide showed no significant differences.

### 3.4. Effect of Vindoline on Levels of Inflammatory Cytokines in the Heart and Kidney Tissues

**Figure 3:**
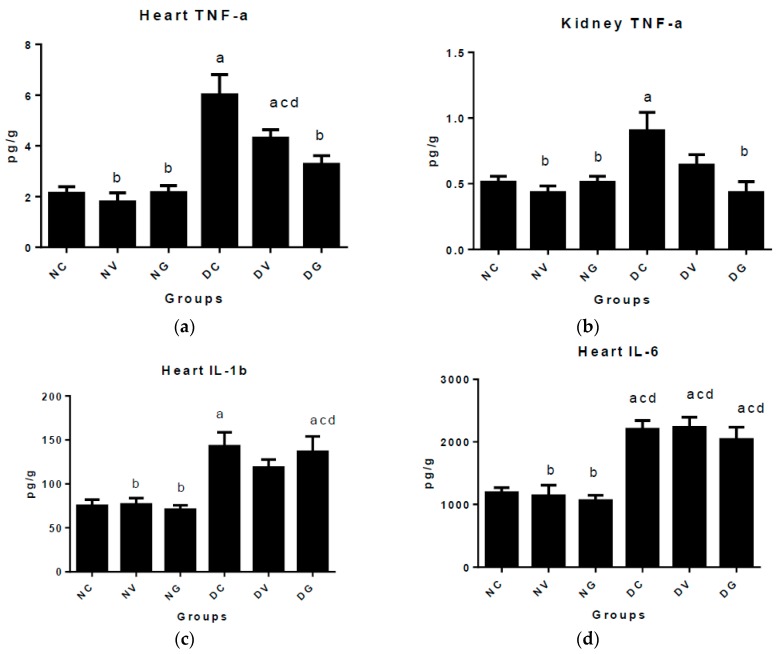

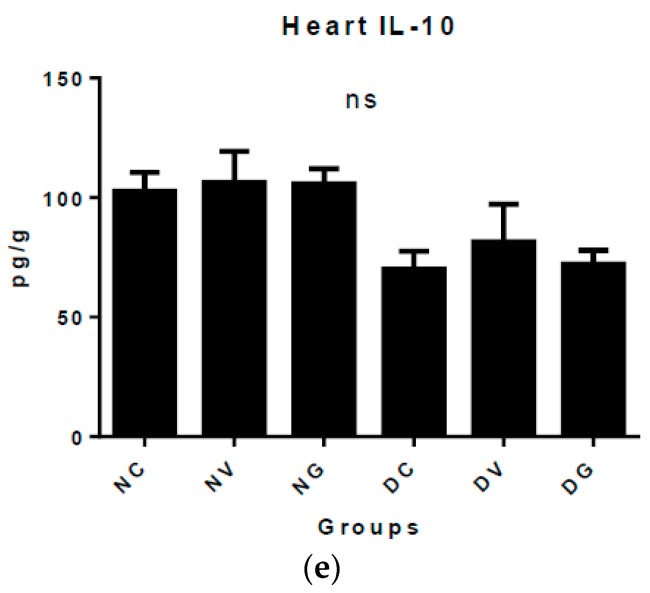
Effect of vindoline on levels of inflammatory cytokines in the heart and kidney tissues after 6-week treatment period represented by the following subfigures; (**a**) heart TNF-α; (**b**) kidney TNF-α; (**c**) heart IL-1β; (**d**) heart IL-6 and (**e**) heart IL-10. Data represented as means ± SEM. ^a^ *p* < 0.05 vs. normal control. ^b^ *p* < 0.05 vs. diabetic control. NC: normal control; NV: normal control treated with vindoline; NG: normal control treated with glibenclamide; DC: diabetic control; DV: diabetic treated with vindoline; DG: diabetic treated with glibenclamide; ns: non-significant vs. all groups.

In the present study, induction of diabetes increased (*p* < 0.05) levels of proinflammatory cytokines TNF-a, IL-1b and IL-6 in rats (Figure 3a–d). The levels of TNF-α in the hearts (Figure 3a) of the untreated diabetic controls and the diabetic group treated with vindoline were elevated significantly when compared to the normal control group. Vindoline in diabetic rats did not significantly reduce the level of TNF-α in the heart tissue when compared to the diabetic control group; however, glibenclamide administration in diabetic rats showed significant reduction of TNF-α. Daily treatment of diabetic rats with vindoline also resulted in no significant changes in the concentrations of TNF-α (Figure 3b) in the kidneys when compared to the normal untreated. Administration of glibenclamide in diabetic rats effectively decreased levels of TNF-a in the kidneys (Figure 3b), the decrease was significant (*p* < 0.05) when compared to the diabetic control group. The levels of IL-1b and IL-6 (Figure 3c,d) in the untreated diabetic controls were elevated significantly when compared to the normal controls. Although the levels of proinflammatory cytokines in both organs were lower in the diabetic group treated with vindoline than in the diabetic control group, the reduction was not significant at (*p* < 0.05). Rats treated with glibenclamide and vindoline displayed significantly increased (*p* < 0.05) IL-6 levels (Figure 3d) in the heart when compared to the normal controls. However, treatment with either vindoline or glibenclamide did not significantly alter the levels of IL-6 in the heart when compared to the diabetic control group.

In addition, treatment of normal and diabetic rats with vindoline or glibenclamide did not significantly alter the levels of the anti-inflammatory cytokine IL-10 in the heart tissue when compared with normal control rats at (*p* < 0.05).

### 3.5. Effect of Vindoline on Oxidative Stress Markers in the Cardiac and Nephron Tissues

**Figure 4:**
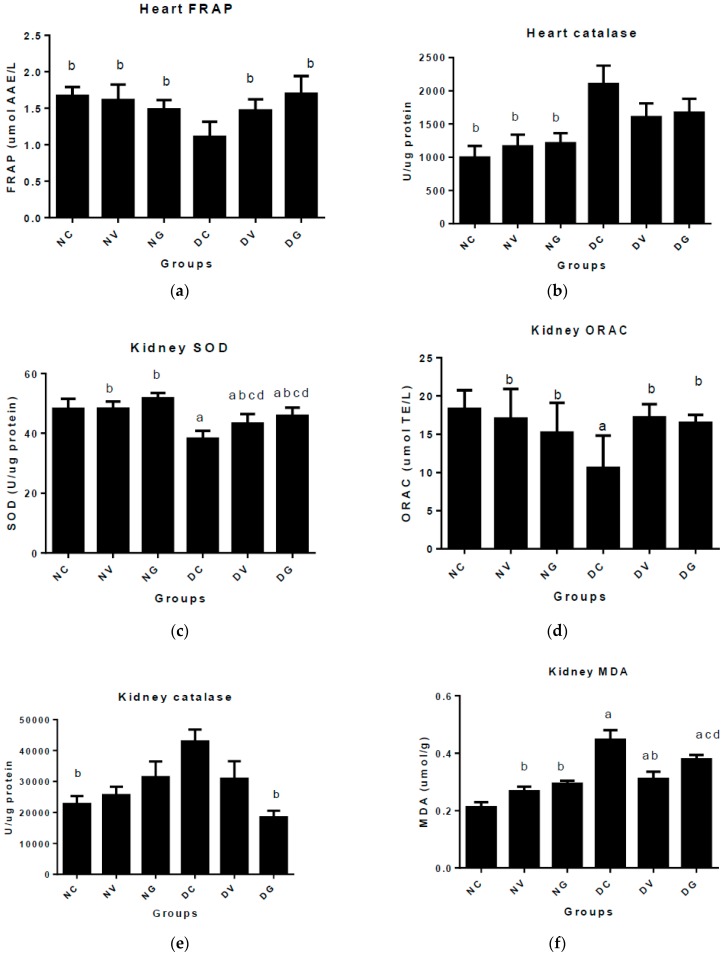
Markers of oxidative stress in the hearts and kidneys of normal and diabetic rats after treatment with vindoline and glibenclamide represented by the following subfigures: (**a**) represents the ferric reducing antioxidant power (FRAP) in the heart tissue; (**b**) catalase activity in the heart tissue; (**c**) SOD activity in the heart tissue; (**d**) oxygen radical absorbance capacity (ORAC) in the kidney tissue; (**e**) catalase activity in the kidney tissue; (**f**) lipid peroxidation (MDA) in the kidney tissue. Data represented as means ± SEM. ^a^ *p* < 0.05 vs. normal control. ^b^ *p* < 0.05 vs. diabetic control. ^c^ *p* < 0.05 vs. normal rats treated with vindoline. ^d^ *p* < 0.05 vs. normal rats treated with glibenclamide. NC: normal control; NV: normal control treated with vindoline; NG: normal control treated with glibenclamide; DC: diabetic control; DV: diabetic treated with vindoline; DG: diabetic treated with glibenclamide.

Figure 4 indicates the antioxidant status in the hearts and kidneys of normal and diabetic rats after treatment with vindoline and glibenclamide for six weeks. The ferric reducing antioxidant power was significantly lower in the cardiac tissue of the untreated diabetic control group when compared to all treatment groups (*p* < 0.05). Treating diabetic rats with vindoline and glibenclamide improved the ferric reducing antioxidant power in the cardiac tissue with no significant differences when compared to all the nondiabetic groups (*p* < 0.05). The activity of catalase in the hearts was determined, the results showed significantly increased activity in the diabetic control group when compared to all nondiabetic groups (*p* < 0.05). Vindoline and glibenclamide administration did not significantly change the activity of catalase when compared to the untreated diabetic control group (*p* < 0.05). The activity of SOD in the kidneys was significantly elevated in all treatment groups when compared to the untreated diabetic control (*p* < 0.05). Treating diabetic rats with vindoline and glibenclamide significantly improved the activity of SOD when compared to the untreated diabetic control group. The oxygen radical absorbance capacity measured in the kidneys was found to be significantly lower in the untreated diabetic controls when compared to all treatment groups. In the diabetic groups treated with vindoline and glibenclamide, the oxygen radical absorbance capacity was significantly improved when compared to the diabetic control group (*p* < 0.05). On the other hand, the catalase activity in the kidney tissue of untreated diabetic controls was significantly elevated when compared to the normal nontreated control group. No significant changes in the activity of catalase in the kidneys were displayed after treating diabetic rats with vindoline when compared to the normal nontreated and diabetic control groups (*p* < 0.05). Interestingly, treating diabetic rats with vindoline significantly reduced the degree of lipid peroxidation in the kidneys when compared to the diabetic control group; nevertheless, the result was still significantly high when compared to the normal untreated control (*p* < 0.05).

### 3.6. Histopathology

**Figure 5:**
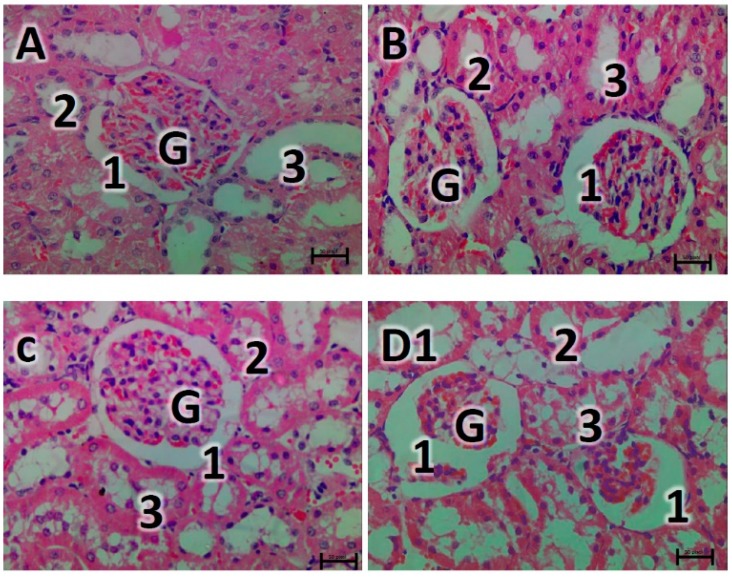

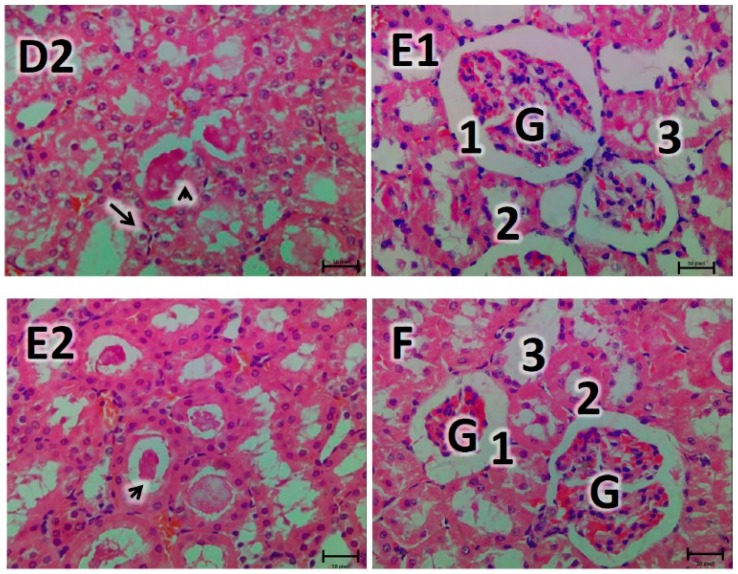
Haematoxylin and eosin stained kidney parenchyma photo micrographs. (**A**) represents: normal control kidney showing the renal cortex with normal glomerulus (G) surrounded by capsule with normal capsular space (1) and normal tubular system distal convoluted tubules (DCT) (2) and normal proximal convoluted tubules (PCT) (3). (**B**,**C**) represent photo micrographs of normal rats treated with vindoline and glibenclamide respectively showing normal renal parenchyma. (**D1**) represents the diabetic control kidney section showing shrunken glomeruli (**G**) with distorted capillaries surrounded by excessive widened Bowman’s space (1), the PCT cells appear to be vacuolated cytoplasm (3). Figure (**D2**) indicates cortical tubules with acidophilic hyaline casts (arrow head). Figure (**E1**) represents the diabetic group treated with vindoline showing glomeruli that are surrounded by narrower Bowman’s space (1), hyaline casts (arrow heads) shown in Figure (**E2**). Figure (**F**) represents the diabetic group treated with glibenclamide showing restored glomerulus (G) with prominent glomerular capillaries, capsular space looks narrow (1).

The sections of the nondiabetic rats represented in Figure 5a–c showed normal kidney histological architecture with no visible signs of lesions. However, the diabetic control group showed severe glomeruli capillary distortion surrounded with a significantly extended capsular space (D1). The renal cortical tubular cells revealed a degree of vacuolation as well as the presence of hyaline casts (D2) indicating tubular degeneration. Treatment of diabetic rats with vindoline and glibenclamide resulted in progressive restoration features which include narrowing of the capsular space and abundant glomerular capillaries (E1 and F). Hyaline casts were also observed in the diabetic rats treated with vindoline (E2).

**Figure 6:**
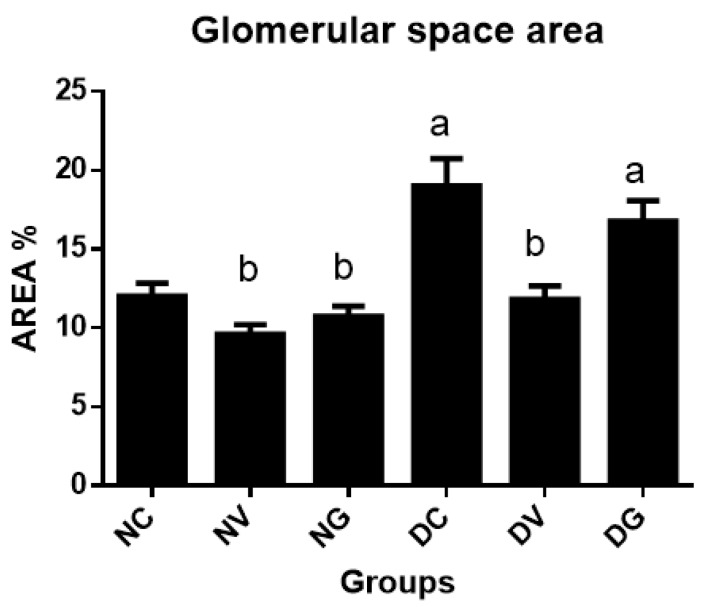
Area occupied by the glomerular space per glomerulus. Data represented as means ± SEM. ^a^ *p* < 0.05 vs. normal control. ^b^ *p* < 0.05 vs. diabetic control. NC: normal control; NV: normal control treated with vindoline; NG: normal control treated with glibenclamide; DC: diabetic control; DV: diabetic treated with vindoline; DG: diabetic treated with glibenclamide.

Figure 6 below represents glomerular space morphometric analysis in the normal and diabetic groups. Significant increase in the capsular space was noted in the diabetic control group when compared to all nondiabetic groups and the diabetic group that was treated with vindoline (*p* < 0.05). No significant changes were seen between the diabetic control and the diabetic group treated with glibenclamide (*p* < 0.05).

### 3.7. Effect of Vindoline on Apoptotic Markers in the Kidney Tissues

**Figure 7:**
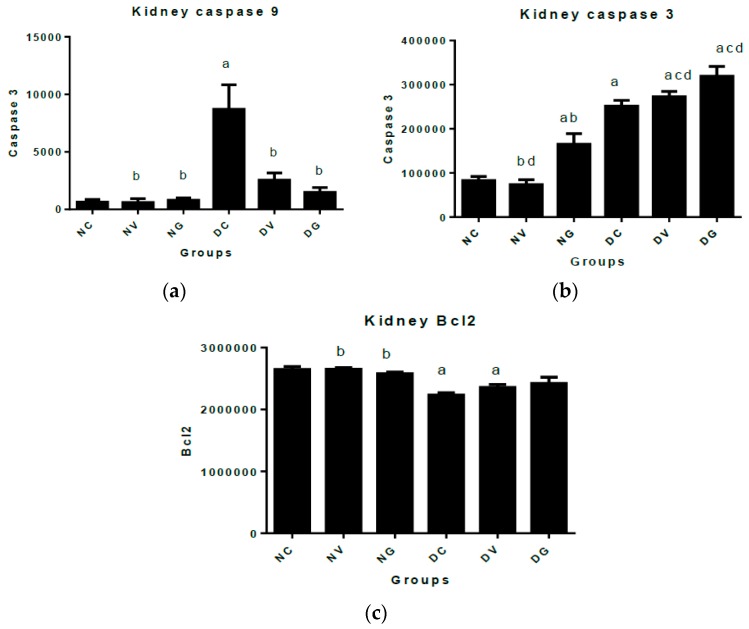

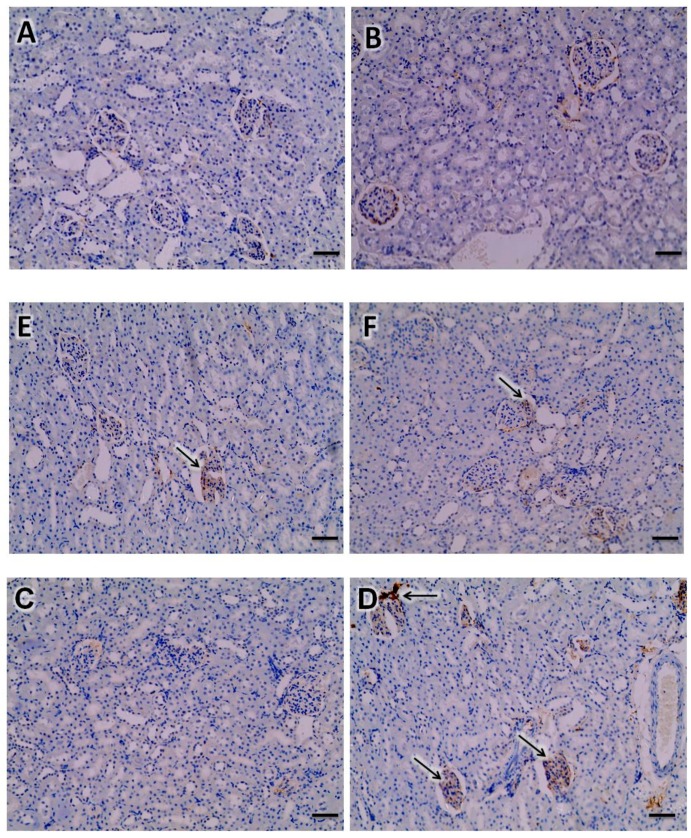

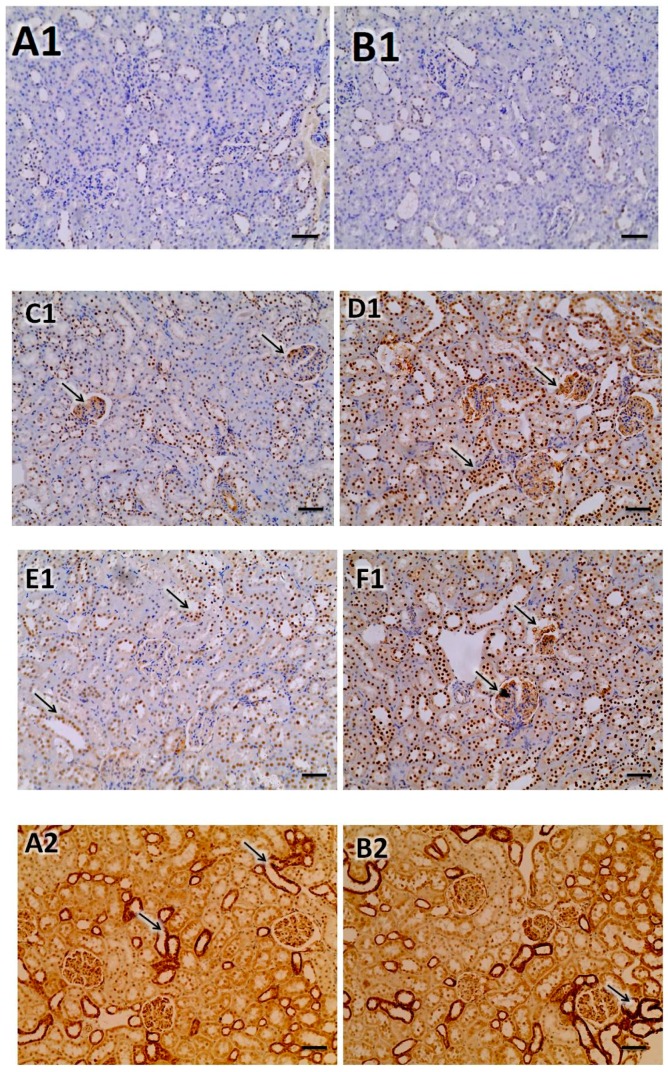

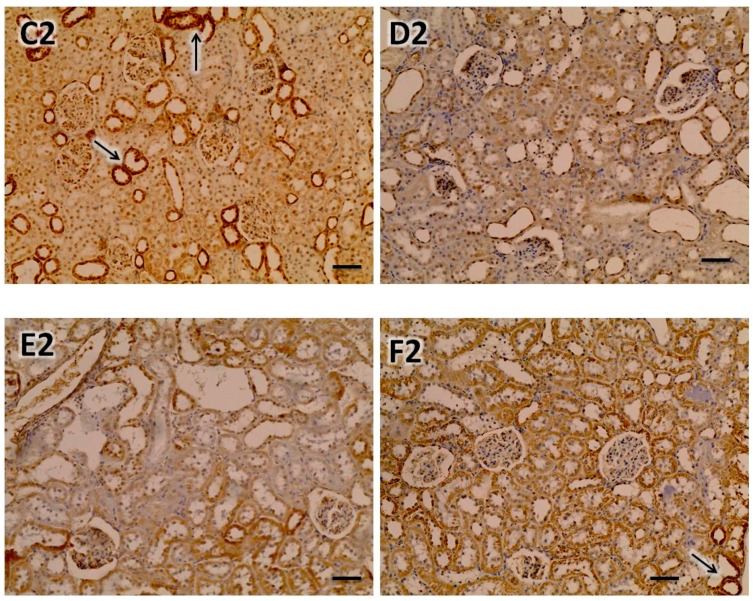
Represent immunohistochemical quantification and immunohistochemical staining in intensities of apoptotic markers. (**a**) Represents immunohistochemical quantification of caspase 9 in the kidney tissue; (**b**) immunohistochemical quantification of caspase 3 in the kidney tissue; (**c**) immunohistochemical quantification of BCL-2 in the kidney tissue. Immunohistochemical photo micrographs indicating staining intensities of different markers are represented as follows: caspase 9 plates (**A**–**F**), caspase 3 (**A1**–**F1**) and BCL-2 (**A2**–**F2**). Arrows represent areas of hyper intensity in varying degrees of the brown stain. Data represented as means ± SEM. ^a^ *p* < 0.05 vs. normal control. ^b^ *p* < 0.05 vs. diabetic control. ^c^ *p* < 0.05 vs. normal rats treated with vindoline. ^d^ *p* < 0.05 vs. normal rats treated with glibenclamide. (NC; A; A1; A2) Normal control; (NV; B; B1; B2) normal control treated with vindoline; (NG; C; C1; C2) normal control treated with glibenclamide; (DC; D; D1; D2) diabetic control; (DV; E; E1; E2) diabetic treated with vindoline; and (DG; F; F1; F2) diabetic treated with glibenclamide. (Scale bar: 50 µm).

Figure 7 below shows immunohistochemical quantification and pictographs of caspase 3, caspase 9 and BCL-2 in the kidney sections of diabetic and normal rats. Caspase 3 was significantly expressed in all diabetic groups when compared to the normal nontreated control group. Vindoline and glibenclamide administration to diabetic rats did not significantly change the expression of caspase 3 when compared to the diabetic control group. On the other hand, normal rats that were treated with glibenclamide showed significant elevated levels of caspase 3 when compared to the normal nontreated controls. The levels of caspase 9 were found to be significantly increased in the diabetic control group when compared to all the groups. Treating diabetic rats with vindoline or glibenclamide prevented the overexpression of caspase 9 when compared to the diabetic control group. However, the levels of BCL-2 in diabetic treated groups were not significantly changed following treatment when compared to the diabetic control group.

## 4. Discussion

Diabetes mellitus is a strong risk factor that leads to the malfunction of the cardiovascular and renal systems resulting in increased mortality [27]. Over the years, there has been an increased research focus on the potentials of plant-derived products in the prevention of diabetes and its complications. This is because plant materials are rich in compounds that may have single or multiple effects in preventing the progression of diabetes and its associated complications [17].

The oral glucose tolerance test is a test that is used to evaluate the body’s ability to utilise glucose [28]. In our present study (Table 1 and Figure 1), we observed a marked reduction in the blood glucose levels in diabetic rats that were treated with vindoline at 90 min post-glucose load and treatment when compared to the diabetic control suggesting improved glucose tolerance. The decrease in blood glucose level may be attributed to insulin secretory effects of vindoline as previously reported [19].

Increase in organ weight (hypertrophy) in relation to the total body weight is common in diabetic rats [29]. The hyperglycaemic environment in diabetes triggers hyper function of the renal and cardiac tissues leading to adverse growth changes of these organs [30]. Alterations in the production of growth factors in the renal tubules have also been linked to kidney hypertrophy [29]. In this study, as indicated in Table 2, the relative weights of the heart and kidney organs of all diabetic groups were found to increase significantly when compared to the nondiabetic rats. Oral administration of vindoline and glibenclamide in diabetic rats did not significantly change the weights of the kidney and heart tissue when compared to the diabetic control group.

Diabetes leads to the progressive deterioration of renal function and eventually resulting in end stage renal disease [31]. Creatinine is a product of the breakdown of creatine phosphate found in the skeletal muscle [32]. Elevated levels of creatinine and urea in blood serum are considered as good/sensitive markers of deranged glomerular filtration, and are thus linked to the severity of kidney injury [33]. The current study observed significantly increased levels of serum creatinine in the untreated diabetic control group when compared to all the groups (Table 2). The observed increase in the serum creatinine levels in the untreated diabetic controls could possibly suggest renal damage and destruction of functioning tubules and nephrons related to DN [34]. Administration of vindoline and glibenclamide to diabetic rats resulted in reduction of serum creatinine levels, suggesting an improved renal function. In addition to improved kidney function, there was a possible decrease in the rate of muscle degradation in the diabetic groups that received treatment [35]. Remarkably, treatment of diabetic rats with vindoline or glibenclamide treatments showed significant reduction of serum urea levels when compared to the diabetic control group (Table 2). These kidney function results may suggest that there was an improvement in the elimination of waste products by the kidneys. However, the levels of serum urea in diabetic groups treated with vindoline and glibenclamide remained significantly higher than that of the normal untreated controls.

Dyslipidaemia is a common complication of diabetes mellitus; it includes increased levels of TC, LDL, VLDL, triglycerides and diminished levels of HDL. Abnormal lipid parameters in DM have been shown to predispose diabetic patients to cardiovascular complications such atherosclerosis, stroke, high blood pressure and coronary heart disease [31]. It has been documented that high circulating levels of LDL and cholesterol are risk factors of pathogenesis of diabetic microvascular and macrovascular complications. LDL acts by transporting cholesterol from the liver to the peripheral organs resulting in lodging of unwanted cholesterol plaques in the endothelium of blood vessels leading to atherosclerosis [36]. In our study, significantly reduced levels of TC, LDL and VLDL were observed in the diabetic groups that were treated with vindoline and glibenclamide when compared to the untreated diabetic control group (Table 3). The decrease in these lipid parameters may possibly indicate the antihyperlipidaemic activities of vindoline and glibenclamide. When compared to the normal nontreated controls, there were no significant differences observed in the levels of TC, LDL and VLDL of diabetic treated groups. We additionally assessed the atherogenic index which is a sensitive marker of potential risk of CVDs development. The diabetic control group exhibited higher atherogenic values when compared to all the groups (Figure 2). Our results indicate that administration of vindoline and glibenclamide to diabetic rats reduced the potential risk of CVDs development when compared to the diabetic controls. The percentage of good cholesterol HDL was determined (Table 3) and the results showed that administering vindoline and glibenclamide significantly restored HDL levels when compared to the diabetic controls, and therefore is likely to reduce the risk of CVD development. Our results are in agreement with those reported by Yao et al. [19]. Islam et al. [37] reported the antihyperlipidaemic effects of *C. roseus*; it is possible that vindoline could be one of the compounds that are responsible for this plant’s reported antihyperlipidaemic effects.

Type 2 DM can be regarded as an immunometabolic disorder in which hyperglycaemia and oxidative stress play pivotal roles in initiating systemic inflammatory responses [38]. Amplified inflammatory responses in DM aggravate endothelial dysfunction which generates vascular complications [39]. Patients with T2DM have been reported to have high levels of circulating inflammatory cytokines which include TNF-α, IL-1β, IL-6, monocyte chemoattractant protein-1 (MCP-1) and C-reactive protein, suggesting an increased risk of tissue injury [40,41]. In our study, induction of diabetes in rats caused significant elevation in the levels of all the proinflammatory cytokines (Figure 3) in the diabetic control group when compared to the normal nontreated control group. Vindoline administration to diabetic rats did not cause significant reduction in the levels of the proinflammatory cytokines in the kidney and heart tissues when compared to the untreated diabetic control group. Glibenclamide treatment in diabetic rats caused significant decrease in the levels of TNF-α in both the heart and kidney tissues when compared to the diabetic controls.

Reactive oxygen species (ROS) are by-products of normal homeostasis; however, in T2DM, hyperglycaemia encourages excessive formation of ROS which ultimately overpowers the endogenous antioxidant machinery leading to oxidative stress [42]. In the presence of oxidative stress, damage to structures such as DNA, lipids, proteins and carbohydrates occurs leading to structural and functional anomalies that initiate the onset and progression of diabetic complications [43]. We assessed the ferric reducing antioxidant power (FRAP) of the heart homogenates (Figure 4). The results showed significantly decreased FRAP in the heart homogenates of untreated diabetic control group when compared to all the groups. This finding suggests the potent antioxidant activity of vindoline and glibenclamide which is important in the prevention of oxidative events that can lead to cardiac injury.

In biological systems, catalase and SOD are the first line of defence against free radicals. SOD acts by dismutating the unstable superoxide anion into oxygen and hydrogen peroxide while catalase converts the latter into water and oxygen [44]. Attenuated functions or insufficient production of these enzymes can result in the accumulation of ROS making the tissues to be susceptible to oxidative damage [43]. Our findings revealed significantly decreased activities of SOD in the kidneys of the untreated diabetic control group when compared to all the groups (Figure 4). Vindoline and glibenclamide treatment in diabetic rats interestingly improved the activity of SOD in the kidneys thereby confirming the possibility of delaying/ prevention of DN development [45]. On the contrary, the activity of catalase in both organs (kidney and heart) of the diabetic control group was found to be elevated when compared to the normal nontreated controls. This finding indicates compensatory responsive mechanisms employed by the antioxidant defence system to overcome oxidative stress [46].

The ORAC assay is a test usually performed to determine the total antioxidant capacity in diabetic models [47]. The ORAC values tend to be low in T2DM signifying uncontrolled hyperglycaemia and oxidative tissue damage [48]. We measured the kidney ORAC in both normal and diabetic rats; we observed significantly low ORAC values in the diabetic control group when compared to the ORAC values of all the groups in this experiment. It was evident that vindoline and glibenclamide boosted the antioxidant defences in the kidneys of diabetic rats. Similar findings were reported by Ayeleso et al. [47] in the erythrocytes of diabetic rats treated rats.

Free radical build-up in biological systems is implicated in the cytotoxic peroxidation of polyunsaturated fatty acids found in cell membranes. MDA are products of lipid peroxidation that are proportional to membrane destruction [41]. In this study, there was increased formation of MDA products in the kidney homogenates of the diabetic control group (Figure 4). The significantly elevated MDA concentration observed in the diabetic control may have occurred as a result of the destruction of the glomerular and renal tubular cell membranes via glucose and lipid oxidation [49]. This finding substantiates the abnormal kidney function observed in this study. On the other hand, oral administration of vindoline to diabetic rats protected the kidney tissue membranes from free radical damage due to the supressed MDA levels noted in comparison to the untreated diabetic control group.

Histopathological examination of the kidney tissue in diabetic control group showed severe loss of the glomerular capillaries, tubular cell degeneration and presence of hyaline casts (Figure 5). These changes observed may be attributed to glomerular hyper filtration, disturbed glucose metabolism and oxidative tissue damage [50,51]. The diabetic group treated with vindoline showed gradual improvement in the structure of the glomerulus and the renal tubules although the renal tubules still had hyaline casts within their lumen. Less injury of the glomerulus and the renal tubules might have been enforced by the antioxidant defence mechanisms [34]. Furthermore, we observed significant dilation of the glomerular capsular space (Figure 6) in the untreated diabetic control group when compared to all groups confirming renal injury due to failed control of hyperglycaemia and increased free radical build-up.

Apoptosis is a normal process whereby cells are programmed by BCL-2 family and caspase proteins to undergo suicide shedding off old, useless or damaged cells [52]. However, several stimuli including changes in DNA expression, low grade inflammation, oxidative, mitochondrial and endoreticulum stress have been implicated in activating abnormal apoptotic signals [41]. BCL-2 super family consists of proteins that are either positive or negative regulators of apoptosis. Bcl-2 has been shown to inhibit mitochondrial apoptosis via inhibiting the insertion of BAX (proapoptotic) into the mitochondrial outer membrane thus preventing the release of cytochrome C [53]. In diminished concentrations of BCL-2; there is successful release of cytochrome C which plays a role in the activation of caspase 9. Caspase 9 in turn initiates apoptosis by activating the effector caspase 3 which executes downstream events of apoptosis which result in cell death [54,55].

Immunohistopathological measurement on the markers of apoptosis in the kidneys of normal and diabetic rats was performed (Figure 7). We observed significantly increased expression of caspase 9 in the untreated diabetic control rats when compared to all treatment groups. This result is in agreement with the findings of Mishra et al. [56] who reported elevated caspase 9 in their type 2 diabetes rat model. High levels of caspase 9 expression suggest the initiation of apoptosis in the glomerulus and renal tubules in response to hyperglycaemia-induced oxidative stress. Interestingly, diabetic rats that received daily treatments of vindoline and glibenclamide showed significantly lower levels of caspase 9 suggesting that vindoline may prevent initiation of apoptosis. However, we did not observe similar findings in caspase 3 expression in both the untreated diabetic controls and diabetic treated groups. No significant changes in the expression of caspase 3 in both diabetic treated groups were found when compared to the untreated diabetic controls; hence the mechanisms of action of vindoline and glibenclamide may not prevent the executional stages of apoptosis.

## 5. Conclusions

Administration of vindoline at a dose of 20 mg/kg body weight could potentially delay the progression of diabetes-related cardiovascular and kidney diseases via improving the antioxidant defence system and delay the initiation of apoptosis. Moreover, vindoline exhibited excellent antihyperlipidaemic activities and could be utilised in the management of microvascular and macrovascular complications associated with diabetes. It is worthwhile to perform detailed toxicity and long term studies to further substantiate these effects.

## Figures and Tables

**Table 1:** Effect of vindoline in nondiabetic and diabetic rats on 2-h oral glucose tolerance test.

Groups	T = 0 h	T = 0.5 h	T = 1 h	T = 1.5 h	T = 2 h	AUC
NC	5.21 ± 0.14	5.31 ± 0.19	5.25 ± 0.13	5.16 ± 0.19	4.76 ± 0.07	621.4
NV	5.28 ± 0.11 ^b^	5.56 ± 0.22 ^b^	5.09 ± 0.19 ^b^	5.06 ± 0.11 ^b^	4.74 ± 0.18 ^b^	622.3
NG	5.68 ± 0.17 ^b^	5.775 ± 0.39 ^b^	4.74 ± 0.36 ^b^	3.86 ± 0.45 ^b^	3.99 ± 0.31 ^b^	576.2
DC	31.89 ± 0.75 ^acd^	31.95 ± 0.72 ^a^	30.31 ± 0.75 ^a^	30.96 ± 0.81 ^a^	27.98 ± 1.30 ^a^	3695
DV	29.05 ± 2.41 ^acd^	28.11 ± 2.19 ^a^	26.48 ± 2.42 ^a^	23.39 ± 2.4 ^ab^	26.08 ± 2.27 ^a^	3166
DG	27.59 ± 1.65 ^acd^	31.48 ± 0.8 ^a^	27.98 ± 1.26 ^a^	26.66 ± 1.97 ^a^	25.39 ± 1.62 ^a^	3375

Data represented as means ± SEM. ^a^ *p* < 0.05 vs. normal control. ^b^ *p* < 0.05 vs. diabetic control. NC: normal control; NV: normal control treated with vindoline; NG: normal control treated with glibenclamide; DC: diabetic control; DV: diabetic treated with vindoline; DG: diabetic treated with glibenclamide. AUC: area under curve.

**Table 2:** Relative organ weights and kidney function parameters following treatment.

Groups	RHW (g)	RKW (g)	Urea (g/L)	Creatinine (mg/dl)
NC	0.28 ± 0.009	0.64 ± 0.02	7.471 ± 0.34	33.72 ± 1.5
NV	0.25 ± 0.005 ^b^	0.61 ± 0.01 ^b^	7.97 ± 0.5 ^b^	37.47 ± 2.5 ^b^
NG	0.25 ± 0.004 ^b^	0.66 ± 0.02 ^b^	6.974 ± 0.5 ^b^	31.14 ± 1.5 ^b^
DC	0.34 ± 0.008 ^a^	1.19 ± 0.03 ^a^	13.66 ± 0.9 ^a^	47.59 ± 4.3 ^a^
DV	0.30 ± 0.005 ^a^	1.07 ± 0.04 ^a^	10.62 ± 0.6 ^ab^	37.24 ± 1.6 ^b^
DG	0.30 ± 0.006 ^a^	1.07 ± 0.04 ^a^	10.82 ± 0.8 ^ab^	37.49 ± 2.3 ^b^

Data represented as means ± SEM. ^a^ *p* < 0.05 vs. normal control. ^b^ *p* < 0.05 vs. diabetic control. NC: normal control; NV: normal control treated with vindoline; NG: normal control treated with glibenclamide; DC: diabetic control; DV: diabetic treated with vindoline; DG: diabetic treated with glibenclamide. RHW: relative heart weight in grams; RKW; relative kidney weight in grams.

**Table 3:** Serum lipid profile for type 2 diabetes mellitus (T2DM)-induced rats after 6 weeks treatment with vindoline.

Groups	TC (g/L)	HDL (%)	TG (g/L)	LDL (g/L)	VLDL (g/L)
NC	0.93 ± 0.07	68.72 ± 2.0	0.54 ± 0.08	0.17 ± 0.04	0.13 ± 0.02
NV	1.3 ± 0.08 ^b^	61.04 ± 1.11 ^bd^	1 ± 0.16 ^b^	0.32 ± 0.04 ^b^	0.18 ± 0.03 ^b^
NG	0.83 ± 0.08 ^b^	74.11 ± 3.54 ^b^	0.54 ± 0.05 ^b^	0.13 ± 0.05 ^b^	0.11 ± 0.01 ^b^
DC	2.83 ± 0.48 ^a^	51.85 ± 3.86 ^a^	2.87 ± 0.6 ^a^	0.75 ± 0.13 ^a^	0.78 ± 0.15 ^a^
DV	1.43 ± 0.12 ^b^	65.58 ± 1.02 ^b^	1 ± 0.2 ^b^	0.3 ± 0.03 ^b^	0.2 ± 0.04 ^b^
DG	1.48 ± 0.18 ^b^	63.10 ± 2.2 ^b^	1.24 ± 0.2 ^b^	0.25 ± 0.04 ^b^	0.24 ± 0.05 ^b^

Data represented as means ± SEM. ^a^ *p* < 0.05 vs. normal control. ^b^ *p* < 0.05 vs. diabetic control. NC: normal control; NV: normal control treated with vindoline; NG: normal control treated with glibenclamide; DC: diabetic control; DV: diabetic treated with vindoline; DG: diabetic treated with glibenclamide; g/L: grams per litre.
